# Sleep, circadian rhythm and gut microbiota: alterations in Alzheimer’s disease and their potential links in the pathogenesis

**DOI:** 10.1080/19490976.2021.1957407

**Published:** 2021-09-14

**Authors:** Yi Li, Lingzhan Shao, Yang Mou, Yan Zhang, Yong Ping

**Affiliations:** a School of Medicine, Imperial College London, London, UK; b Bio-X Institutes, Key Laboratory for the Genetics of Developmental and Neuropsychiatric Disorders (Ministry of Education), Shanghai Jiao Tong University, Shanghai, China; c Shanghai Mental Health Center, School of Medicine, Shanghai Jiao Tong University, Shanghai, China

**Keywords:** Sleep, circadian rhythm, gut microbiota, alzheimer’s disease, animal models, inflammation

## Abstract

In recent years, emerging studies have observed gut microbiota (GM) alterations in Alzheimer’s disease (AD), even in individuals with mild cognitive impairment (MCI). Further, impaired sleep and circadian patterns are common symptoms of AD, while sleep and circadian rhythm disruption (SCRD) is associated with greater β-amyloid (Aβ) burden and AD risk, sometimes years before the clinical onset of AD. Moreover, reports have demonstrated that GM and its metabolites exhibit diurnal rhythmicity and the role of SCRD in dampening the GM rhythmicity and eubiosis. This review will provide an evaluation of clinical and animal studies describing GM alterations in distinct conditions, including AD, sleep and circadian disruption. It aims to identify the overlapping and distinctive GM alterations in these conditions and their contributions to pathophysiology. Although most studies are observational and use different methodologies, data indicate partial commonalities in GM alterations and unanimity at functional level. Finally, we discuss the possible interactions between SCRD and GM in AD pathogenesis, as well as several methodological improvements that are necessary for future research.

## Introduction

Alzheimer’s disease (AD) is a degenerative central nervous system (CNS) disorder, characterized by a progressive onset of neurocognitive symptoms, including amnesia, aphasia, disorientation, etc.^1^ While the etiology of AD remains largely unknown, AD is generally featured by the deposition of β-amyloid (Aβ) and the formation of neurofibrillary tangles of tau protein in CNS.

The human body harbors a large variety of microorganism communities which intensively interact with host and each other through direct contacts or metabolites.^2^ It has long been postulated that human gut microbiota (GM), the collection of all microorganism communities in the human digestive tract, holds great significance to human health and disease.^3,4^ However, not until recently have we been able to investigate their composition and function with the advances in DNA sequencing and metagenomic analysis techniques.^5^ Moreover, brain-gut-axis (BGA), which studies the interactions between GM and CNS, has gained significant attention in recent years. There is much evidence showing altered GM composition in several neurological diseases, including Parkinson’s disease (PD) and autism spectrum disorder (ASD).^6–8^ Changes in GM composition and richness have also been observed in AD patients and individuals with mild cognitive impairment (MCI),^9,10^ suggesting a potential role of GM dysbiosis in AD pathogenesis.

Several neurodegenerative diseases including AD, PD and Huntington disease (HD) have been implicated with sleep disturbance and circadian rhythm dysfunction.^11^ While sleep and circadian rhythm disruption (SCRD) are usually recognized as the consequences of these diseases, studies have reported the existence of sleep disorders long before the onset of AD and PD, even by decades.^12–15^ Moreover, growing evidence indicates that sleep disturbance and circadian rhythm misalignment may contribute to neuroinflammation, low Aβ clearance efficacy, increased concentration of reactive oxygen species (ROS), compromised blood-brain-barrier (BBB) and GM dysbiosis.^16–18^ However, the present work revealed the correlation between SCRD and AD, but not causality, and further work is needed to resolve this issue.

**Figure 1.**
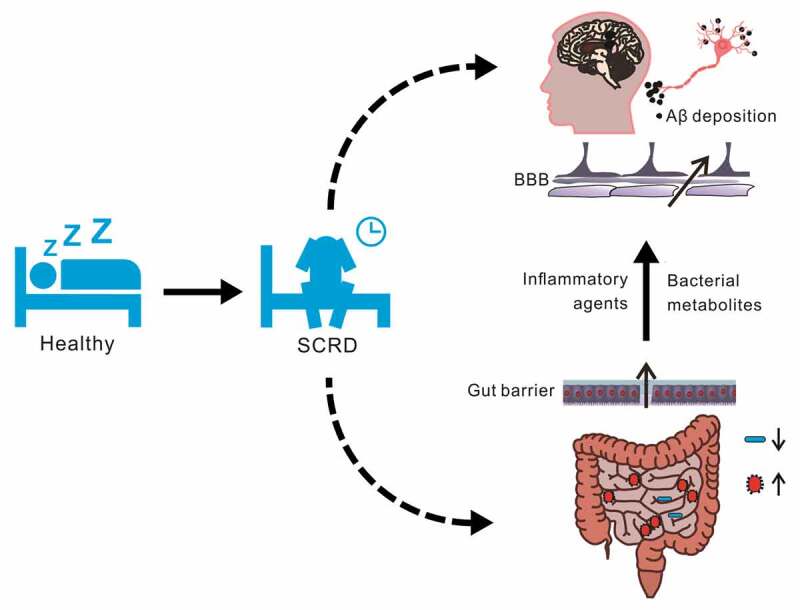
A hypothetical model of linking SCRD, GM and AD pathogenesis. SCRD caused by sleep disorders or working night shift impairs brain functions in many ways, one of which acts through GM. SCRD leads to GM dysbiosis, with increase in pathobionts and decrease in beneficial bacteria. In the bottom of the figure, blue color represents symbionts such as beneficial bacteria, while red color represents pathobionts. Integrated gut barrier and BBB normally block pathogens such as bacteria metabolites from entering the brain. However, GM dysbiosis caused by SCRD disrupt gut barrier and BBB by degrading mucin and releasing proinflammatory agents and neurotoxic metabolites. These pathological changes can cause aberrant neuroinflammation, and subsequently lead to Aβ deposition and AD onset

Studies in the last few decades have long examined common determinants of the human GM, including diet, medicine and stress.^19,20^ Recent findings suggest a novel role of sleep and circadian rhythm in shaping and modulating the composition of GM.^21^ However, to the best of our knowledge, no reviews to date have considered the possible contributions of synergistic interactions between SCRD and GM dysbiosis to the pathogenesis of AD. In this review, we first present recent studies that examined the GM alterations in AD and SCRD. We summarize those findings and compare the GM changes at both compositional and functional levels across studies. We observe commonalities in GM alterations of individual bacteria and unanimous changes at functional level between AD and SCRD conditions. Therefore, we discuss possible interactions between SCRD and GM, which contribute to AD onset by inducing peripheral and central inflammation (Figure 1). We reason that this is achieved through various pathways including disrupted gut barrier integrity, compromised blood-brain barrier (BBB), decreased short-chain fatty acids (SCFAs) production and increased pro-inflammatory metabolites.

## GM and AD

The role of microorganisms in the pathogenesis of AD was initially proposed by Alois Alzheimer, the first describer of this progressive neurodegenerative disorder.^22^ After decades of insufficient research, there has been a resurgence of interests in this hypothesis, largely owing to a growing body of evidence from clinical and animal tests. Several kinds of infectious agents such as bacteria, fungi, virus and protozoa that are highly associated with AD have been reviewed elsewhere.^1,23–25^ In this part, we focus on GM alterations, probiotic and antibiotic treatments, and fecal microbiota transplantation (FMT) in both AD patients and models.

### GM alterations in AD: from clinical and animal literature

Recent clinical observations have found significant GM alterations in both AD and MCI patients. Here, we summarize the alterations of GM composition in AD patients compared to controls in Table 1 (top).^9,10,26–28^ In addition, animal models are also used in other studies, and the relevant findings are summarized in Table 1 (bottom).^29–36^ Note that transgenic mice including APP/PS1, SAMP8, 5xFAD and their derivatives were the most frequently used AD models.^37^ Substances such as D-galactose, Aβ protein and lipopolysaccharide (LPS) were also used in several studies to induce AD pathology.^38^

**Table 1.** Summary of studies investigating GM alteration in AD

Reference	Participant/animal model	GM profiling method	Higher or lower bacterial taxa in AD patients/AD animal models		Other major findings
Human study					
26	43 AD patients and 43 age- and gender-matched HC Location: China	16S rRNA gene seq V3-V4 region	↑	Family: Enterococcaceae, Lactobacillaceae	
				Genus: *Subdoligranulum*	
				Species: *Ruminococcus gnavus*	
			↓	Family: Lachnospiraceae, Bacteroidaceae, Veillonellaceae	
				Genus: *Lachnoclostridium, Bacteriodes*	
9	30 AD patients, 30 MCI patients, and 30 age- and gender-matched HC Location: China	16S rRNA gene seq V3-V4 region	↑	Family: Lachnospiraceae, Streptococcaceae, Erysiopelotrichaceae, Coriobacteriaceae, Lactobacillaceae, Bifidobacteriaceae	- Similar alteration of gut and blood microbiota in AD and MCI - Increased blood *Staphylococcus, Pseudomonas*, and *Escherichia* in AD and MCI vs. HC - *Dorea, Blautia*, and *Escherichia* as risk factors for AD
				Genus: *Akkermansia, Blautia, Dorea, Eggerthella, Streptococcus, Bifidobacterium, Lactobacillus*	
			↓	Family: Alcaligenaceae, Bacteroidaceae, Porphyromonadaceae, Pasteurellaceae, Rikenellaceae	
				Genus: *Alistipes, Bacteroides, Butyricimonas, Haemophilus, Parabacteroides*	
10	33 AD patients, 32 aMCI patients, and 32 age- and gender-matched HC Location: China	16S rRNA gene seq V3-V4 region	↑	Family: Enterobacteriaceae, Veillonellaceae	- Progressive enrichment of Enterobacteriaceae distinguishes AD from aMCI and HC - Elevated bacterial secretion system and LPS biosynthesis
			↓	Family: Clostridiaceae, Lachnospiraceae, Ruminococcaceae	
				Genus: *Blautia, Ruminococcus*	
27	25 AD patients and 25 age- and gender-matched HC Location: USA	16S rRNA gene seq V4 region	↑	Family: Bacteroidaceae, Rikenellaceae, Gemellaceae	
				Genus: *Blautia, Bacteroides, Alistipes, Bilophila, Gemella, Phascolarctobacterium*	
			↓	Family: Ruminococcaceae, Bifidobacteriaceae, Clostridiaceae, Peptostreptococcaceae, Mogibacteriaceae, Turicibacteraceae	
				Genus: *Bifidobacterium, Dialister, Clostridium, Turicibacter, Adlercreutzia*	
28	40 Amy+ patients, 33 Amy- patients, and 10 HC Location: Italy	Microbial DNA qPCR Assay Kit	Amy+ *vs*. HC		- Escherichia and Shigella correlate with pro-inflammatory IL-1β, NLRP3 and CXCL2 - Eubacterium rectale correlates with anti-inflammatory IL-10
			↑	Genus: *Escherichia, Shigella*	
			↓	Species: *Eubacterium rectale, Bacteroides fragilis*	
Animal study					
29	Female APP/PS1 mice Control: female WT mice Age: 3, 6 and 24 months	16S rRNA gene seq V1-V3 region	↑	Family: Erysipelotrichaceae	- Progressive GM shift in AD mice at 3 months
				Genus: *Sutterella*	
			↓	Family: Rikenellaceae	
				Genus: *Ruminococcus, Oscillospira*	
30	Male SAMP8 mice Control: male SAMR1 mice Age: 6 months	16S rRNA gene seq V3-V4 region	↑	Genus: *Alistipes, Akkermansia, norank_f__Lachnospiraceae, Odoribacter, Streptococcus, Rikenella, Butyricicoccus*	- Altered GM structure with decreased fermentation capacity - Dysregulated lipid, carbon and pyruvate metabolism
			↓	Genus: *Prevotella, Parasutterella, Butyrivibrio, Eubacterium, Ruminococcus, norank_f__S24_7*,	
31	Male APP/PS1 mice Control: male WT mice Age: 6 months	16S rRNA gene seq V3-V4 region	↑	Family: Verrucomicrobiaceae, Desulfovibrionaceae, Staphylococcaceae, Corynebacteriaceae	- Alleviated AD pathology in AD mice after FMT from WT mice - Increased level of butyrate in FMT-treated AD mice
				Genus: *Akkermansia, Staphylococcus, Desulfovibrio, unclassified_f__Erysiopelotrichaceae*,	
			↓	Family: S24_7, Prevotellaceae, Enterococcaceae	
				Genus: *Faecalibaculum, Ruminococcaceae UCG-01, Alloprevotella, Enterococcus*	
32	Male SAMP8 mice Control: male SAMR1 mice Age: 7 months	16S rRNA gene seq V3-V5 region	↑	Genus: *uncultured Bacteroidales bacterium*	- Decreased spatial learning and memory function in WT pseudo GF mice after FMT from AD mice
			↓	Family: Clostridiales vadinBB60 group, Family XIII, Christensenellaceae, Ruminococcaceae, Desulfovibrionaceae, Deferribacteraceae	
				Genus: *Mucispirillum, Serratia, Subdoligranulum, Ruminiclostridium, Coprococcus, Oscillibacter*	
33	Male APP/PS1 mice Control: male WT mice Age: 1, 3, 5–6, 8–12 months	16S rRNA gene seq V3-V4 region	↑	Family: Erysiopelotrichaceae, Verrucomicrobiaceae	- Lower level of SCFAs in feces and brain of AD mice - Disrupted intestinal structure
				Species: *Desulfovibrio C21_c20*	
			↓	Genus: *Ruminococcus, Butyricicoccus*	
				Species: *Butyricicoccus pullicaecorum*	
34	Male APP/PS1 mice Control: male WT mice Age: 3, 6 and 8 months	16S rRNA gene seq V3-V4 region	↑	Family: Helicobacteraceae, Desulfovibrionaceae, Coriobacteriaceae	- Impaired spatial learning and increased Aβ burden in AD mice
				Genus: *Odoribacter, Helicobacter*	
			↓	Genus: *Prevotella, Ruminococcus*	
36	Male/female APP/PS1 mice Control: male and female WT mice Age: 8 months	16S rRNA gene seq V3-V4 region	↑	Family: Enterobacteriaceae, Staphylococcaceae, Lachnospiraceae, Rikenellaceae	- More severe Aβ pathology induced by FMT from AD mice
				Genus: *Staphylococcus*	
			↓	Family: Bifidobacteriaceae, Coriobacteriaceae, Bacteroidaceae, Prevotellaceae, Turicibacteraceae, Akkermansiaceae	
				Genus: *Bifidobacterium, Prevotella, Turicibacter, Desulfovibrio, Akkermansia*	
35	Female ADLP^APT^ mice Control: female WT mice Age: 8 months	16S rRNA gene seq	↑	Family: Prevotellaceae, Rikenellaceae	- Damaged gut barrier and chronic inflammation - Attenuated cognitive impairment and Aβ burden in AD mice after FMT from WT mice
				Genus: *Prevotella, Paraprevotella*	
			↓	Family: Lactobacillaceae, Turicibacteraceae, Desulfovibrionaceae, S24-7	
				Genus: *Lactobacillus, Turicibacter, Desulfovibrio*	

Note: HC = healthy control, aMCI = amnestic mild cognitive impairment, WT = wild type, FMT = fecal microbiota transplantation, GF = germ free, ↑ = higher, ↓ = lower.

**Figure 2.**
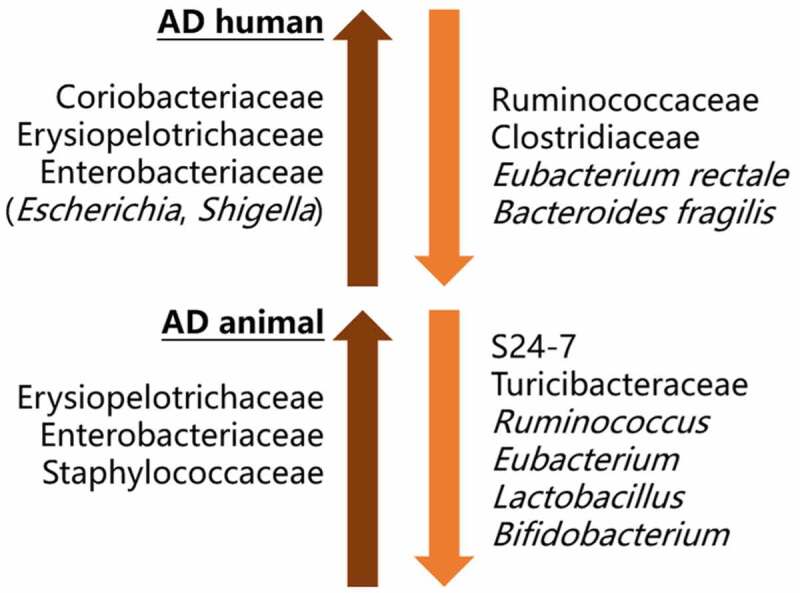
A diagram showing GM compositional changes in AD studies. Increased pro-inflammatory taxa like Erysiopelotrichaceae and Enterobacteriaceae were observed in both AD patients and AD animal models. *Escherichia* and *Shigella* of Enterobacteriaceae, which have long been proposed to contribute to series of gastrointestinal diseases, could disrupt the integrity of epithelial cell and lead to leaky gut. Anti-inflammatory *Eubacterium* and SCFA-producing *Ruminococcus* were decreased in AD. Two probiotic taxa *Lactobacillus* and *Bifidobacterium* have been proven to restore cognitive function and ameliorate Aβ pathology in AD animals

It has been suggested that α-diversity analysis and Firmicutes/Bacteroidetes (F/B) ratio, two frequently used criteria in microbiome analysis, are not reliable in investigating the association between GM alteration and PD.^6,39^ Interestingly, we also found inconsistent results of α-diversity, F/B ratio and GM changes at high phylogenetic rank (e.g., phylum, class and order level) in both AD and SCRD studies. The findings showed better concordance at higher taxonomic resolution. Therefore, GM alterations at family, genus and species level are presented in the following tables (Tables 1–5). Generally, we have identified higher level of pathobionts and lower level of beneficial bacteria in both AD patients and animals (Figure 2).

**Table 2.** Summary of studies investigating GM intervention and AD

Reference	Participant/animal model	Treatment	Main findings (Exp *vs*. Con)
Probiotic supplement			
51	AD patients Exp: AD patients + probiotic milk Con: AD patients + normal milk	Duration: 12 weeks Probiotic milk contained *Lactobacillus acidophilus, Lactobacillus casei, Bifidobacterium bifidum*, and *Lactobacillus fermentum*	- ↑ cognitive function - ↑ insulin and lipid metabolism
54	AD patients Exp: data after taking Omnibiotic Stress Repair Con: baseline data before probiotic treatment	Duration: 4 weeks Omnibiotic Stress Repair contained 9 strains from *Lactococcus, Lactobacillus*, and *Bifidobacterium*	- ↑ *Faecalibacterium prausnitzii* - ↑ tryptophan metabolism and serum kynurenine
55	Female App^NL-G-F^ mice Exp: AD mice + VSL#3 Con: AD mice + vehicle (water)	Duration: 8 weeks VSL#3 contained 8 strains of lactic acid-producing bacteria	- ↓ intestinal inflammation and gut permeability
52	Male 3xTg-AD mice Exp: AD mice + SLAB51 Con: AD mice + vehicle (water)	Duration: 4 months SLAB51 contained 9 live probiotic strains	- ↓ cognitive impairment and brain damage - ↓ pro-inflammatory cytokines - ↓ Aβ deposition in brain
56	Male ddY mice + intra-hippocampal Aβ injection Exp: AD mice + probiotic supplement/acetate Con: AD mice + vehicle (water)	Duration: starting 2 days before Aβ injection Probiotic supplement: living, heat-killed or fragmented *Bifidobacterium breve* A1	- ↓ cognitive impairment - Altered gene expression in hippocampus - ↑ plasma acetate by *B. breve* A1 - Partially attenuated behavioral deficit by non-viable *B. breve* A1 and acetate
57	Male Wistar rats + intra-hippocampal Aβ injection Exp: AD rats + probiotic supplement Con: AD rats + vehicle (water)	Duration: 8 weeks Probiotic supplement: *Lactobacillus acidophilus, Lactobacillus fermentum, Bifidobacterium lactis*, and *Bifidobacterium longum*	- ↑ spatial memory - ↓ Aβ deposition in brain - ↓ oxidative stress response
58	Male Sprague-Dawley rats Exp: (1) rats + antibiotic, (2) rats + antibiotic + probiotic Con: rats + vehicle (water)	Duration: 41 days Antibiotic: ampicillin Probiotic: *Lactobacillus fermentum* NS9	- Disrupted GM in (1) and normalized GM in (2) - ↓ colon inflammation in (2) *vs*. (1) - ↑ spatial memory in (2) *vs*. (1)
Antibiotic treatment			
59	Male APP/PS1 mice Exp: AD mice + ABX treatment Con: AD mice + vehicle (water)	Duration: post-natal day 14 to day 21 ABX contained 9 antibiotics	- Altered GM composition - ↓ Aβ deposition in the brain - ↓ glial reactivity at Aβ plaque - ↓ neuroinflammation
60	Male APP/PS1 mice Exp: AD mice + ABX treatment Con: AD mice + vehicle (water)	Duration: lifespan ABX contained 9 antibiotics	- Altered GM composition - ↓ Aβ deposition in the brain - ↓ neuroinflammation and reactive gliosis at Aβ
61	5xFAD mice Exp: AD mice + ABX treatment Con: AD mice + vehicle (water)	Duration: 5 months ABX contained ampicillin, streptomycin and colistin	- ↓ GM abundance - ↓ infiltration of pro-inflammatory Th1 cells and M1 cells into the brain
62	APPPS1-21 mice Exp: (1) male + ABX, (2) female + ABX Con: male/female + vehicle (water)	Duration: lifespan ABX contained kanamycin, gentamicin, colistin, metronidazole and vancomycin	- Sex-specific gut microbiota alteration - (1): ↑ anti-inflammatory cytokines, ↓ Aβ, and ↓ phagocytic microglial at Aβ - (2): ↑ pro-inflammatory cytokines, no change of Aβ deposition, and ↑ phagocytic microglial at Aβ
63	Male 5xFAD mice Exp: AD mice + ABX treatment Con: AD mice + vehicle (water)	Duration: 2 months ABX contained vancomycin, cefoxitin, gentamicin, and metronidazol	- ↑ ceca size and weight - ↓ level of hippocampal Aβ - ↑ cognitive function
64	Male APPPS1-21 mice Exp: (1) AD mice + ABX, (2) AD mice + individual ABX Con: AD mice + vehicle (water)	Duration: lifespan ABX contained kanamycin, gentamicin, colistin, metronidazole, and vancomycin	- ↑ ceca size and altered GM composition - ↓ Aβ deposition only in (1)
Germ-free animal			
36	APP/PS1 mice Exp: GF AD mice Con: conventionally raised AD mice	GF mice: embryos were washed with Invitrogen and transferred to GF pseudo-pregnant mice	- ↓ Aβ level and Aβ deposition - ↓ neuroinflammation - ↑ Aβ-degrading enzyme
65	Female APP/PS1 mice Exp: (1) SPF AD mice, (2) GF AD mice Con: (3) SPF WT mice, (4) GF WT mice		- Altered GM composition in (1) *vs*. (3) - ↓ cognitive function in (1)(2) *vs*. WT - ↑ Aβ and neuroinflammation in (1) *vs*. (2) and (3) - ↑ MAPK signaling pathway in (1) *vs*. (2) and (3)
63	Male 5xFAD mice Exp: GF AD mice Con: SPF AD mice	GF mice were generated through embryo transfer	- ↑ ceca size and weight - ↓ Aβ and neuroinflammation - ↑ cognitive function - ↑ Aβ uptake by microglial
FMT and co-housing			
35	Female ADLP^APT^ mice Exp: AD mice + WT FMT Con: AD mice + vehicle (water)	Duration: 16 weeks FMT: oral gavage	- ↓ cognitive impairment - ↓ Aβ, tau pathology, and glial activity - ↓ expression of inflammation-related genes
36	GF APP/PS1 mice Exp: (1) GF AD mice + AD FMT, (2) GF AD mice + WT FMT Con: GF AD mice + vehicle (water)	FMT: oral gavage	- ↑ overall Aβ level in (1) and (2) - Higher level of increased brain Aβ42 in (1) *vs*. (2)
61	WT mice Exp: WT mice co-housed with AD mice Con: WT mice separately housed with AD mice	Duration: 7 months	- ↓ discriminating learning - Similar GM and cytokine expression to AD mice - ↑ infiltrating Th1 cells into brain
61	(1) WT mice + Aβ injection + AD FMT (2) AD mice + WT FMT (3) WT mice + Aβ injection + GV-971-treated AD FMT	FMT: oral gavage	- (1) ↑ Th1 cells and ↓ Th2 cells in brain - (2) ↓ Th1 cells in brain - (3) ↓ Th1 cells in brain
31	Male APP/PS1 mice Exp: AD mice + WT FMT Con: AD mice + vehicle (water)	FMT: oral gavage	- ↓ neuroinflammation - ↓ Aβ deposition and tau phosphorylation - ↓ GM dysbiosis and cognitive deficits
32	Male pseudo GF WT mice Exp: (1) GF mice + SAMP8 FMT, (2) GF mice + SAMP1 FMT Con: GF WT mice + vehicle (water)	Duration: 14 days FMT: oral gavage	- ↓ cognitive function in pseudo GF mice - Restored GM composition in (2) not (1) - ↑ cognitive function in (2) not (1)
62	ABX-treated male APPPS1-21 mice Exp: ABX-treated AD mice + AD FMT Con: ABX-treated AD mice + vehicle (water)	Duration: lifespan FMT: oral gavage	- ↑ Aβ plaque burden - GM profile similar to AD mice - Microglial morphologies similar to AD mice

Note: Exp = experimental group, Con = control group, ABX = antibiotic cocktail, GF = germ-free, SPF = specific pathogen-free, ↑ = increase, ↓ = decrease.

**Table 3.** Summary of studies examining the impact of sleep disturbance on GM and correlation between sleep quality and bacterial taxa

Reference	Participant/animal model	GM profiling method	GM alterations by sleep disturbance/correlated with poor sleep quality			Other major findings
Human study						
71	9 healthy males Partial SD *vs*. NS Location: Sweden	16S rRNA gene seq V4 region	↑		Family: Coriobacteriaceae, Erysiopelotrichaceae	- Increased insulin resistance and fasting insulin level
72	28 healthy adults PSQI for sleep measuring Location: USA	16S rRNA gene seq V4 region	+		Genus: *Prevotella*	
			-		Family: Lachnospiraceae	
					Genus: *Blautia, Ruminococcus*	
73	37 adults aging from 50 to 85 PSQI for sleep measuring Location: USA	16S rRNA gene seq	-		Phylum: Verrucomicrobia, Lentisphaerae	- Better Stroop and Color-Word performance were associated with better sleep quality
74	22 healthy males Actiwatch for sleep measuring Location: USA	16S rRNA gene seq V4 region	+		Family: Lachnospiraceae	
					Genus: *Blautia, Lachnospiraceae UCG-004, Oribacterium*	
			-		Genus: *Lachnospiraceae ND3007*	
Animal study						
75	Male C57BL/6 J mice Chronic SF *vs*. NS	16S rRNA gene seq V4 region	↑		Family: Lachnospiraceae, Ruminococcaceae	- Increased food intake, VWAT, inflammation, insulin resistance, and gut permeability - Enhanced inflammation in GF mice after FMT from SF mice
			↓		Family: Lactobacillaceae, Bifidobacteriaceae	
76	Male C57BL/6 J mice Short SD *vs*. NS	16S rRNA gene seq V3-V5 region	↑		Family: Lachnospiraceae	- Subtle GM alteration by short period of SD
					Genus: *Moryella*	
			↓		Genus: *Oxobacter*	
77	Male Wistar-Kyoto rats SF *vs*. NS	16S rRNA gene seq V4 region	↑		Genus: *Escherichia, Shigella, Enterococcus, Lachnospiraceae UCG-008*	- Increased mean arterial pressure
			↓		Genus: *Butyrivibrio, Oscillospira, Eubacterium, Dorea*	
					Species: *Eubacterium ruminantium*	
78	Male C57BL/6 N mice SD *vs*. NS	16S rRNA gene seq V4 region	↓		Family: Bifidobacteriaceae, Lactobacillaceae, Turicibacteraceae	- Reduced fecal bile acid and triterpenoids
					Genus: *Bifidobacterium, Lactobacillus, Turicibacter*	
79	Sprague Dawley rats Acute SF (ASF) *vs*. NS Chronic SF (CSF) *vs*. NS	Distal ileum (D), cecum (C), and proximal colon (P) samples 16S rRNA gene seq	ASF	↑	Family: Enterobacteriaceae (D), S24-7 (D), Ruminococcaceae (C)	- Increased microbial invasion - Altered intestinal structure but not gut barrier integrity - Increased KC/GRO level
					Genus: *Oscillospira* (C), *Bacteroides* (C), *Prevotella* (C)	
				↓	Family: Lactobacillaceae (D)	
					Genus: *Lactobacillus* (P)	
			CSF	↑	Family: Staphylococcaceae (D), Clostridiaceae (D)(P), Erysipelotrichaceae (P), Ruminococcaceae (P)	
					Genus: *Prevotella* (P), *Clostridium* (P)	
				↓	Family: Lactobacillaceae (D)	
80	Male Wistar rats Paradoxical SD *vs*. NS	16S rRNA gene seq	↑		Genus: *Parabacteroides, Ruminococcus, Aggregatibacter, Phascolarctobacterium*	- Depression-like behavior - Increased CRH, ACTH, and CORT and pro-inflammatory cytokines IL-6, TNF-α, and CRP - Decreased arginine, proline, and pyruvate metabolism
			↓		Genus: *Akkermansia, Oscillospira*	

Note: NS = normal sleep, SD = sleep deprivation, SF = sleep fragmentation, PSQI = Pittsburgh Sleep Quality Index, FMT = fecal microbiota transplantation, GF = germ free, ↑ = increase, ↓ = decrease, + = positively correlated, – = negatively correlated.

**Table 4.** Summary of research studying the impact of circadian rhythm disruption on GM

Reference	Participant/animal model	GM profiling method	GM alterations by circadian rhythm disruption			Other major findings
Human study						
87	10 healthy males Night shift *vs*. day shift Location: Turkey	16S rRNA gene seq	↑		Family: Coriobacteriaceae, Erysipelotrichaceae, Prevotellaceae, Lachnospiraceae	
					Genus: *Dorea, Coprococcus*	
					Species: *Ruminococcus torques, Ruminococcus gauvreauii*	
			↓		Species: *Faecalibacterium prausnitzii*	
68	2 healthy individuals After jet lag *vs*. before jet lag	16S rRNA gene seq V1-V2 region	↑		Phylum: Firmicutes	- Human GM showed diurnal oscillation - FMT from jet-lagged individual into GF mice caused weight gain and body fat accumulation
			↓		Phylum: Bacteroidetes	
88	22 healthy adults Acute sleep-wake cycle shift After shift vs. before shift Location: China	16S rRNA gene seq V4 region	↑		Family: Pasteurellaceae, Fusobacteriaceae	- Acute sleep-wake cycle shift had limited impact on GM
					Genus: *Dialister, Escherichia, Shigella*	
			↓		Family: Peptostreptococcacea, Desulfovibrionaceae	
					Genus: *Ruminococcaceae UCG-013*	
Animal study						
89	Male C57BL/6 J mice Inverted light (IN) *vs*. LD	16S rRNA gene seq V4 region	↑		Genus: *Barnesiella, Clostridium, Lactobacillus*	- Increased weight gain, inflammation, and insulin resistance - Disrupted gut barrier by fecal water of IN mice
			↓		Genus: *Turicibacter*	
90	Male C57BL/6 J mice LL *vs*. LD	16S rRNA gene seq	↑		Species: *Ruminococcus torques*	- Increased LPS synthesis and decreased SCFAs and indole metabolism - Disrupted gut barrier integrity
			↓		Genus: *Subdoligranulum*	
					Species: *Lactobacillus johnsonii, Eubacterium plexicaudatum*	
91	Male rats LL *vs*. LD DD *vs*. LD	16S rRNA gene seq V3-V4 region	LL	↑	Family: Erysiopelotrichaceae, Bacteroidaceae, Prevotellaceae, Lactobacillaceae	- Increased anxiety and activity
					Genus: *Blautia, Prevotella, Lactobacillus, Faecalibacterium*	
				↓	Family: Ruminococcaceae, Porphyromonadaceae	
					Genus: *Parabacteroides*	
			DD	↑	Family: Erysiopelotrichaceae, Prevotellaceae, Lactobacillaceae	- Decreased activity - Decreased DA and NE in urine
					Genus: *Blautia, Prevotella, Lactobacillus, Faecalibacterium*	
				↓	Family: Ruminococcaceae, Porphyromonadaceae	
					Genus: *Parabacteroides, Bacteroides, Ruminococcus*	
68	WT mice Jet lag *vs*. LD	16S rRNA gene seq V1-V2 region	↑		Family: Prevotellaceae, Rikenellaceae	- Mice GM exhibited diurnal oscillation - Disrupted diurnal rhythmicity of GM by jet lag
			↓		Family: Christensenellaceae, Anaeroplasmataceae	
					Genus: *Lactococcus, Dorea, Lactobacillus, Ruminococus*	

Note: LD = normal light cycle, LL = constant light, DD = constant dark, FMT = fecal microbiota transplantation, GF = germ free, ↑ = increase, ↓ = decrease.

**Table 5.** Summary of the trend of GM alteration in AD and SCRD

Implication in health and disease		Taxonomic level		Trend of GM alteration		
		Family	Genus/Species	AD	SD	CRD
Human study						
Beneficial bacteria	Producing SCFAs Promoting mucin expression Anti-inflammatory	Akkermansiaceae	*Akkermansia*	//	N/A	N/A
	Inhibiting inflammation and infection	Bacteroidaceae	*Bacteroides fragilis* (NTBF)	↓(S*)	N/A	N/A
	Producing GABA, acetate, and lactate	Bifidobacteriaceae	*Bifidobacterium*	//	N/A	N/A
	Producing SCFAs	Clostridiaceae		↓(F**)	N/A	N/A
	Producing butyrate Anti-inflammatory	Eubacteriaceae	*Eubacterium rectale*	↓(S*)	N/A	N/A
	Producing SCFAs	Lachnospiraceae	*Blautia*	//	//	N/A
	Producing GABA, lactate, and amino acid	Lactobacillaceae	*Lactobacillus*	//	N/A	N/A
	Producing butyrate Anti-inflammatory	Ruminococcaceae	*Faecalibacterium*	↓(F**)	N/A	↓(G*)
	Producing SCFAs		*Ruminococcus*		↓(G*)	N/A
Controversial taxa	Producing propionate Degrading mucin Increasing gut permeability	Lachnospiraceae	*Dorea*	↑(G*)	N/A	↑(F*, G*)
			*Ruminococcus gauvreauii*	N/A	N/A	↑(S*)
			*Ruminococcus gnavus*	↑(S*)	N/A	N/A
			*Ruminococcus torques*	N/A	N/A	↑(S*)
Pathobionts	Positively correlated with IBD	Coriobacteriaceae		↑(F*)	↑(F*)	N/A
	Producing LPS, bacteria Aβ, and exotoxin Damaging gut barrier Pro-inflammatory	Enterobacteriaceae	*Escherichia*	↑(F*, G*)	N/A	↑(G*)
			*Shigella*	↑(F*, G*)	N/A	↑(G*)
	Highly immunogenic Pro-inflammatory	Erysiopelotrichaceae		↑(F*)	↑(F*)	↑(F*)
		Prevotellaceae	*Prevotella*	N/A	↑(G*)	↑(F*)
Animal study						
Beneficial bacteria	Producing SCFAs Promoting mucin expression Anti-inflammatory	Akkermansiaceae	*Akkermansia*	//	↓(G*)	N/A
	Inhibiting inflammation and infection	Bifidobacteriaceae	*Bifidobacterium*	↓(G*)	↓(F**, G*)	N/A
	Producing butyrate Anti-inflammatory	Eubacteriaceae	*Eubacterium plexicaudatum*	↓(G*)	N/A	↓(S*)
			*Eubacterium ruminantium*		↓(G*, S*)	N/A
	Producing SCFAs	Lachnospiraceae	*Blautia*	//	//	//
	Producing butyrate		*Butyrivibrio*	↓(G*)	↓(G*)	N/A
	Producing GABA, lactate, and amino acid	Lactobacillaceae	*Lactobacillus*	↓(G*)	↓(F****, G**)	//
	Producing SCFAs	Ruminococcaceae	*Ruminococcus*	↓(G****)	↑(F***, G*)	↓(F**, G**)
	Negatively correlated with IBD	S24-7		↓(F***)	N/A	N/A
	Negatively correlated with IBD, ASD	Turicibacteraceae	*Turicibacter*	↓(F**, G**)	↓(F*, G*)	↓(G*)
Controversial taxa	Producing propionate Degrading mucin Increasing gut permeability	Lachnospiraceae	*Dorea*	//	↑(F***)	//
			*Ruminococcus torques*	N/A	N/A	↑(S*)
Pathobionts	Producing LPS, bacteria Aβ, and exotoxin Damaging gut barrier Pro-inflammatory	Enterobacteriaceae	*Escherichia*	↑(F*)	↑(F*, G*)	N/A
			*Shigella*		↑(F*, G*)	N/A
	Highly immunogenic Pro-inflammatory	Erysiopelotrichaceae		↑(F**)	↑(F*)	↑(F**)
		Prevotellaceae	*Prevotella*	//	↑(G**)	↑(F***, G**)
	Producing bacterial Aβ and toxin Pro-inflammatory	Staphylococcaceae	*Staphylococcus*	↑(F**, G**)	↑(F*)	N/A

Note: ↑ = increase, ↓ = decrease, // = both increase and decreased were reported, N/A = not reported, F = family level, G = genus level, S = species level, * = number of study.

The pro-inflammatory taxa *Escherichia* and *Shigella* of Enterobacteriaceae have long been proposed to contribute to series of gastrointestinal diseases.^10^ Increased level of *E. coli* LPS has also been detected in the postmortem brain samples of AD patients.^40^ The exotoxin of *Escherichia* and *Shigella* could disrupt the integrity of epithelial cell further leading to leaky gut and facilitates the translocation of bacteria into the blood.^41^ *E. coli* along with several gram-negative bacteria possess systems for producing bacterial Aβ which is able to penetrate intestinal barrier and BBB and initiate cross-seeding in the CNS.^42,43^ In addition to *Escherichia*, bacterial Aβ producing systems have also been found in *Staphylococcus*, highlighting its potential role in contributing to AD pathogenesis.^44^ Although *Staphylococcus* was not detected in human fecal sample, its higher abundance was found in the blood of AD patients.^9^ Studies have reported that strains of *Ruminococcus gnavus* which belong to the family Lachnospiraceae use terminal mucin glycans to degrade mucus layer of intestinal barrier.^45^ Increased level of *Ruminococcus gnavus* has been associated with inflammatory bowel disease, suggesting the potential role of *Ruminococcus gnavus* in promoting inflammation.^46^

The two families Ruminococcaceae and Clostridiaceae, major SCFA-producing taxa in mammalian GM, have been reported to be decreased in various metabolic and neurodegenerative diseases.^47^ The relative abundance of Ruminococcaceae was found to be positively correlated with higher Mini-mental State Examination (MMSE) and Montreal Cognitive Assessment (MoCA) scores, which indicates better cognitive functions.^10^ Lower level of anti-inflammatory taxa *Eubacterium rectale* and *Bacteroides fragilis* along with increased pro-inflammatory cytokines such as IL-1β, NLRP3 and CXCL2 have been also detected in AD patients.^28^ *Lactobacillus* and *Bifidobacterium* are two common probiotic taxa capable of producing neurotransmitter gamma-amino butyrate (GABA) whose metabolism has been reported to be disrupted in AD patients.^48^ *Lactobacillus* and *Bifidobacterium* play an important role in protecting intestinal cells and inducing anti-inflammatory responses.^49,50^ Studies have shown that probiotic treatment using strains of *Lactobacillus* and *Bifidobacterium* was able to ameliorate symptoms associated with AD.^51,52^

### GM interventions restore the progression of AD

**Figure 3.**
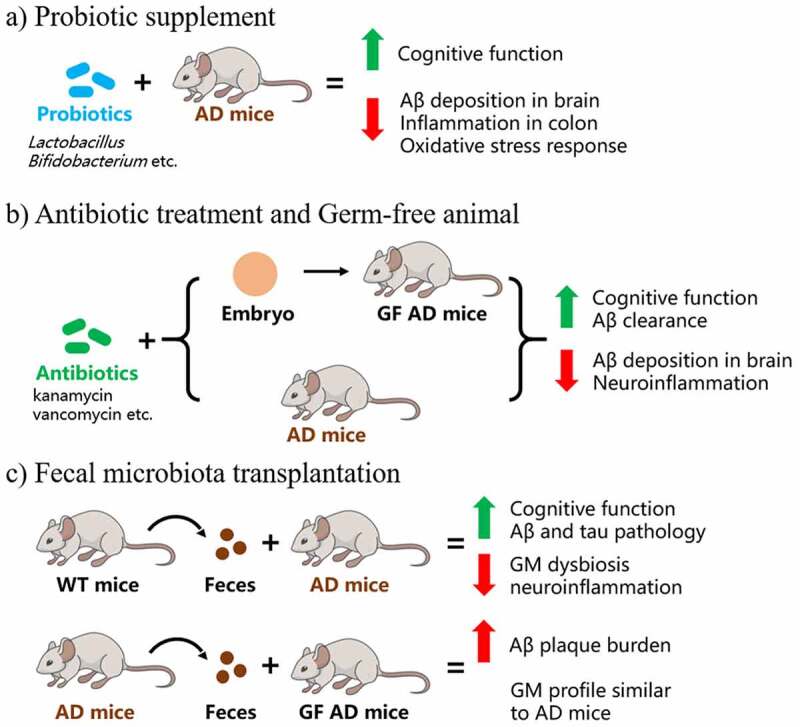
GM intervention studies in AD animal models. (a) Probiotic supplement study: AD mice feed with probiotic strains of *Lactobacillus* and *Bifidobacterium* showed reversed cognitive dysfunction, decreased Aβ deposition in brain and lower level of colon inflammation. (b) Antibiotic treatment and germ-free (GF) animal study: antibiotic treated embryo was transferred to pseudo-pregnant mice to generate GF mice. Both GF AD mice and AD mice feed with antibiotic display improved cognitive function, increased Aβ clearance and alleviated neuroinflammation. (c) Fecal microbiota transplantation (FMT) study: FMT from healthy wild-type (WT) donor could restore GM dysbiosis, ameliorate Aβ and tau pathology, and downregulate neuroinflammation in AD mice, whereas GF AD mice receiving FMT from AD mice show aggravated Aβ burden and GM profile similar as observed in AD mice

As stated above, most studies focusing on GM and AD presented correlations but not causal relationships. While it remains an open question in the field,^53^ several studies have begun to demonstrate how GM affect AD pathology by showing the beneficial effects through GM intervention in animal models, including probiotic supplement,^51,52,54–58^ antibiotic treatment,^59–64^ germ-free (GF) animals^36,63,65^ and fecal microbiota transplantation (FMT).^31,32,35,36,61,62^ These successful trials support the role of GM dysbiosis in contributing to AD pathogenesis and progression and suggest potential benefits of GM modulation for AD treatment (Table 2) (Figure 3).

## Sleep, circadian rhythm and GM

Although human gut ecosystem maintains rather resilient, perturbation by antibiotics, high-fat food and stress could damage intestinal homeostasis.^3,66^ These key determinants of GM have been studied extensively over the past decades, but the role of sleep and circadian rhythm in regulating GM was underestimated.^67^ Recent studies have shown that human GM display diurnal oscillation at both compositional and functional levels.^68^ It has been suggested that SCRD may lead to GM dysbiosis through several indirect ways, including disrupting the rhythmic fluctuation of GM, activating the HPA axis, increasing food and energy intake, decreasing physical activity and damaging gut barrier integrity.^21,69,70^ In this part, we summarize recent progress regarding the correlation between SCRD and GM dysbiosis as well as how SCRD impacts GM (Tables 3, 4). Like the findings in AD, increased pathobionts and decreased beneficial bacteria were identified in SCRD conditions in both human and animal models.

### Sleep disturbance and GM alterations

GM alterations in human and animal models caused by sleep disturbance or related to sleep quality are presented in Table 3 (top)^71–74^ and Table 3 (bottom),^75–80^ respectively. To date, only a few studies explored the effects of sleep impacting on GM in humans, restricting their focus on the association between specific bacterial taxa and sleep quality based on Pittsburgh sleep quality index (PSQI) or sleep physiology. Two studies compared the GM of individuals after short-term sleep deprivation with baseline data collected before deprivation.^71,81^ But their findings are largely inconsistent, likely owing to distinct experimental designs and several uncontrolled variables, including daily dietary and energy intake of the subjects. Therefore, few commonalities in GM changes can be concluded from human studies. In contrast, multiple animal-based experimental studies that focus on the impacts of long-term sleep deprivation and fragmentation on GM composition have been conducted, with largely identical results of GM alterations.

#### Increased bacterial taxa by sleep disturbance

In humans, partial sleep deprivation and poor sleep quality resulted in more abundant Erysiopelotrichaceae, Prevotellaceae and Coriobacteriaceae at family level (Table 3, top). Sleep deprivation and fragmentation in animals contributed to GM dysbiosis featured by increased Ruminococcaceae, Lachnospiraceae, Erysiopelotrichaceae, Enterobacteriaceae and Staphylococcaceae at family level, and *Ruminococcus, Prevotella, Escherichia* and *Shigella* at genus level (Table 3, bottom).

Prevotellaceae is also an immunogenic bacterial taxon highly coated by IgA.^82^ It has also been suggested that species of Prevotellaceae could induce intestinal inflammation, slow the development of mucus layer and are involved in various intestinal diseases including IBD and colitis.^83^ Note that although sleep disturbance increased abundance of Ruminococcaceae and Lachnospiraceae in murine subjects, it is mainly due to increased food-intake as both families are highly fermentative bacteria utilizing the plant-derived fiber and polysaccharides in chow food.^75^

#### Decreased bacterial taxa by sleep disturbance

In human studies, a decline in the relative abundance of *Ruminococcus* is correlated with poor sleep quality (Table 3, top). In animal subjects, Lactobacillacea, Bifidobacteriaceae, Turicibacteraceae at both family and genus level, together with *Eubacterium* and *Akkermansia* at genus level, exhibited significant decrease after sleep deprivation (Table 3, bottom).

Eubacteriaceae along with Clostridiaceae, Lachnospiraceae and Ruminococcaceae are important SCFAs producers of mammalian GM.^49^ The SCFA butyrate plays an important role in maintaining gut barrier and regulating immune responses toward anti-inflammatory status.^84^ The genus *Eubacterium* makes significant contribution to butyrate production since *Eubacterium rectale* makes up about 13% of the clostridial cluster XIVa.^49^ Therefore, loss of *Eubacterium* caused by sleep disturbances could lead to a decline in butyrate level and disrupt the integrity of gut barrier. It has been found that the SCFA-producing taxon *Akkermansia* can successfully mitigate the development of obesity and diabetes, protect gut barrier integrity and stimulate anti-inflammatory responses.^85^

### Circadian rhythm disruption and GM alterations

In addition to sleep loss, circadian rhythm disruption is also receiving increasing attention, given the increased prevalence of altered sleep-wake cycle and jet lag, which are largely due to working night shift and traveling across time zones. Aberrant light exposure, high fat diet, alcohol consumption and irregular eating behavior have been found to induce circadian misalignment.^86^ Numerous studies have indicated a link between circadian rhythm disruption with higher risk of pathological conditions including obesity, cardiovascular diseases and neurodegenerative diseases. The diurnal oscillation of human GM is partially controlled by central clock,^68^ indicating the regulatory roles of circadian in GM eubiosis. Thus, we summarized recent studies focusing on the effects of circadian rhythm disruption on GM components in Table 4.^68,87–91^

#### Increased bacterial taxa by circadian rhythm disruption

The GM of human after undergoing shift work or jet lag exhibited increased abundance of Erysiopelotrichaceae, Prevotellaceae and Lachnospiraceae at family level, *Dorea* at genus level, and *Ruminococcus torques* and *Ruminococcus gauvreauii* at species level (Table 4, top). In murine models, circadian rhythm disruption (mainly achieved by altering light-dark cycles) resulted in an increase of Erysiopelotrichaceae and Prevotellaceae at family level, *Prevotella* at genus level and *Ruminococcus torques* at species level, largely consistent with observations in humans (Table 4, bottom).

*Dorea, Ruminococcus torques* and *Ruminococcus gauvreauii* utilize glycoside hydrolases to breakdown mucus layer and produce propionate.^92^ Despite their SFCA-producing capacity, increased abundance of mucolytic bacteria has been associated with disrupted gut barrier and inflammatory bowel diseases.^93^ Studies have suggested the role of *Dorea* spp. in inflammation through the promotion of IFNγ production and mucin degradation.^84,94^ Significantly abundant pathobiont *Ruminococcus torques* has been found in patients with ulcerative colitis (UC) and CD.^93^ *Ruminococcus gauvreauii* has been found to be positively correlated with pro-inflammatory parameters in rats with fatty liver.^95^

#### Decreased bacterial taxa by circadian rhythm disruption

In human studies, circadian disruption led to decreased levels of genus *Faecalibacterium* and species *Faecalibacterium prausnitzii* (Table 4, top). Ruminococcaceae at both family and genus level, *Turicibacter* at genus level and *Eubacterium plexicaudatum* at species level were decreased in animal studies after the disruption of light-dark cycles (Table 4, bottom).

*Faecalibacterium* was the only diminished bacterial taxa caused by circadian rhythm disruption at genus level. *Faecalibacterium prausnitzi*, the sole species of genus *Faecalibacterium*, is one of the most abundant bacteria in human GM representing more than 5% of bacterial population in intestine.^96^ It acts as an important SCFA butyrate producing taxon, similar to other members in *Ruminococcaceae* family.^97^ Moreover, studies have reported a negative association of *Faecalibacterium prausnitzi* with various inflammatory bowel diseases including UC and CD, suggesting that it could be a health indicator.^96^

## Linking GM, sleep, circadian and AD

### GM and AD – causal or coincidental?

What is the role of GM dysbiosis in AD? It remains debatable whether GM dysbiosis plays as causal or merely consequential role in AD. Recently, studies have started to support the idea that GM dysbiosis precedes the onset of AD and even contributes to AD pathogenesis. Li *et al*. found that AD and MCI groups had distinct GM compositions from healthy controls in both fecal and blood samples, largely consistent with a previous report by another group.^9,10^ These findings provide a new perspective that GM dysbiosis starting at early MCI is a developing process with the cumulation and depletion of specific bacterial taxa. Studies of GM intervention in AD including probiotic supplement, antibiotic treatment, germ-free animals and FMT further reinforced the causal role of GM dysbiosis in AD pathogenesis.

What causes GM dysbiosis before the onset of AD? Human GM is determined by multiple factors including early life exposure, medical intervention, diet, stress, sleep and circadian rhythm.^21^ Many studies have associated these factors with GM eubiosis, and their potential impacts on AD pathogenesis. A recent paper proposed a perspective that diet-induced GM dysbiosis plays a role in the pathogenesis of AD.^44^ Multiple reviews summarized GM alterations in AD and SCRD, respectively, but no reviews to date have systematically analyzed the patterns of GM changes in AD and SCRD simultaneously, or made a hypothesis linking SCRD, GM dysbiosis and AD.

### Linking SCRD to AD through GM dysbiosis

**Figure 4.**
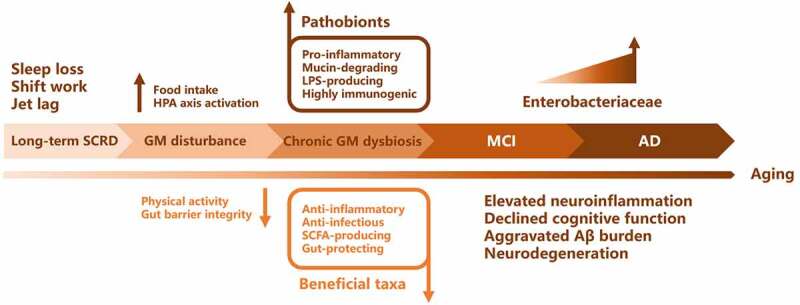
Time-line for the development of AD via SCRD-induced GM dysbiosis. Long-term SCRD (e.g., insomnia, fragmented sleep, night shift work and frequent traveling between time zones) leads to chronic alteration of GM with overabundant pathobionts and reduced beneficial bacteria. GM dysbiosis disrupts gut barrier integrity and facilitates the invasion of pathogens and their metabolite (e.g., LPS, exotoxins and bacterial Aβ). These pro-inflammatory agents induce inflammation responses and compromise BBB structure, leading to neuroinflammation and the onset of early MCI. As MCI develops, progressive enrichment of pathobionts such as Enterobacteriaceae further exacerbate neuroinflammation, cognitive dysfunction and Aβ burden, which in the end contribute to the pathogenesis of AD

As shown in the previous parts, GM alterations were observed in AD, sleep and circadian disruption, respectively. Reports have also indicated that GM alterations might contribute to AD pathogenesis.^98,99^ Studies which have been reviewed elsewhere have shown that SCRD was associated with greater Aβ burden and AD risk, sometimes decades before the clinical onset of AD.^16^ Therefore, we hypothesize that the interactions between SCRD and GM lead to GM dysbiosis indirectly; as a consequence, chronic systematic and neuro-inflammation and Aβ deposition occur, together with a plethora of metabolic and immunogenic responses that may finally contribute to the onset of AD (Figure 4).

First, we check the uniformity in GM alterations and their potential contributions to health and disease under AD and SCRD conditions. We compared the GM alterations and their potential roles (beneficial bacteria, pathobionts or controversial taxa) in a taxonomic view under distinct conditions: AD, sleep and circadian disruption (Table 5). We observe higher abundance of highly immunogenic Erysiopelotrichaceae at family level in both human and rodents in each condition, but most other changes in individual bacteria were inconsistent between human and rodent (Table 5), which may be caused by the differences in GM components between these two species.^100^ Thus, when analyzing the overlapping of GM alterations in different conditions, we conduct separate evaluations in humans and rodents. In humans, SCFAs-producing Ruminococcaceae at family or genus level is shown to be significantly lower in either condition, whereas highly immunogenic bacteria including Erysiopelotrichaceae and Coriobacteriaceae at family level are shown to be significantly higher in each condition. Most other GM components are inconsistent between different conditions, sometimes due to no relevant data available at present (Table 5). In animal models, similar trends are observed in several bacteria individuals between different conditions. For example, beneficial bacteria including Lactobacillaceae, Bifidobacteriaceae, Turicibacteraceae and Lachnospiraceae at family and/or genus level are significantly decreased in AD, sleep disturbance and/or circadian disruption, and other parts of pathobionts are uniformly increased, with the exception of Ruminococcaceae. As stated above, the increase in Ruminococcaceae during sleep disturbance was probably due to aberrant food intake.

Next, we elucidate the potential role of GM dysbiosis in the development of AD by providing the evidence of how GM interventions, including probiotics, antibiotics, germ-free treatment and FMT, restore cognitive functions and alleviate AD pathology (Table 2) (Figure 3). Although various factors modulate GM composition, emerging evidence has indicated that SCRD could disturb GM and lead to GM dysbiosis. Most human studies merely investigated the correlation between SCRD and GM dysbiosis, while animal studies provided more insights into GM alterations under different SCRD conditions such as sleep deprivation, sleep fragmentation and circadian rhythm reversal. Studies have also revealed several possible mechanisms underlying how SCRD contributes to GM dysbiosis, including increased food intake, decreased physical activity, activation of HPA axis and compromised gut barrier integrity, and this topic has been reviewed elsewhere.^21,101^

**Figure 5.**
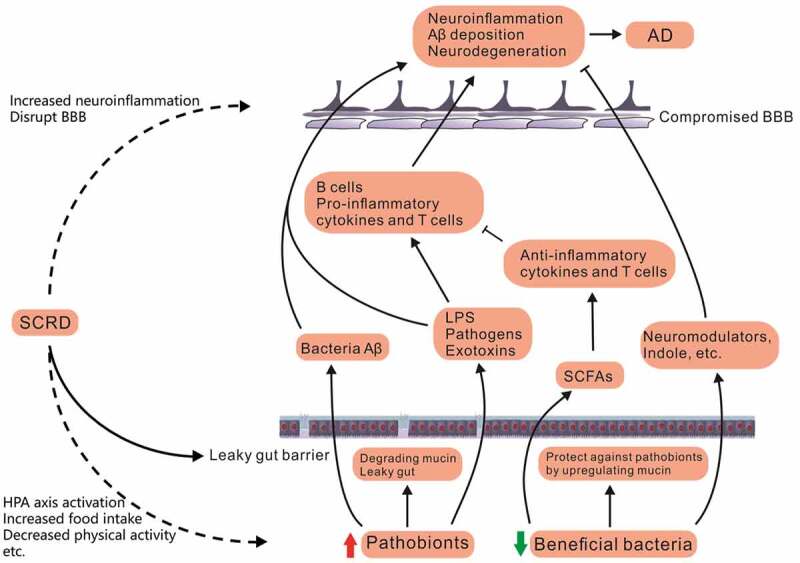
Schematic diagram of how SCRD contributes to AD pathogenesis through GM dysbiosis. SCRD, such as sleep deprivation, sleep fragmentation and jet lag, disrupts gut homeostasis with increased pathobionts (e.g., Enterobacteriaceae, Erysiopelotrichaceae and Prevotellaceae) and decreased beneficial bacteria (e.g., Eubacteriaceae, Ruminococcaceae and other SCFA-producing taxa). On one hand, pathobionts could damage gut barrier and cause leaky gut through the degradation of mucus layer. Pathogens and their metabolites induce pro-inflammatory responses and lead to increased BBB permeability. Bacteria-derived Aβ and LPS invade CNS and are associated with neuroinflammation and Aβ pathology. On the other hand, the compromised functions of beneficial bacteria (e.g., inhibiting infection, promoting mucin expression, producing neuromodulators and anti-inflammation SCFAs) are overwhelmed by overabundant pathobionts. Thus, the elevated neuroinflammation and aggravated Aβ burden facilitate the onset of AD

Finally, we evaluate the specific roles of each individual bacteria and its potential contributions to health and disease. Intriguingly, dysfunctions mediated by the GM alterations are ideally unanimous in AD and SCRD conditions. Both AD and SCRD are associated with more abundant pathobionts leading to pro-inflammation and lower SCFAs, and less level of anti-inflammatory, SCFA-producing, and gut barrier-protecting bacteria (beneficial bacteria) (Table 5). These analyses demonstrate that GM dysbiosis caused by SCRD is largely consistent with the ones in AD, supporting our hypothesis that SCRD may contribute to AD partially by impacting on GM (Figure 5).

## Future directions

In this review, we intend to summarize and evaluate the commonalities and distinctiveness of GM alterations in different conditions including AD, sleep disruption and circadian rhythm misalignment. Although data implied commonalities in these conditions, there were also condition-specific changes in certain species. Significantly, heterogeneity of methodologies applied for genetic material extraction, DNA sequencing, the lifestyle of subjects and methods for data analysis could compromise the results among different studies and lead to inconsistency, which could be expected in human studies. We suggest that further work is needed to specify the alteration of GM at species and even strain level, and incorporate metabolic and functional analysis to reveal possible mechanisms linking GM dysbiosis and diseases using standardized experimental design and data analysis.

### Phylogenetic analysis of GM needs to be conducted at a high taxonomic resolution

Studies have implicated that GM can be altered at lower taxonomic level without achieving alteration at high taxonomic level.^39^ For example, Firmicutes and Bacteroidetes are the two largest bacterial phyla of the mammalian gastrointestinal tract, and their ratio (F/B) was commonly used in GM analysis.^102^ However, reviews have reported inconsistent changes in F/B ratio across a series of neurodegenerative diseases and metabolic disorders, making F/B ratio a debatable and controversial criterion.^6,99,103,104^ In agreement with our findings, one review summarizing the GM alterations in patients with PD found that, at high taxonomic ranks like phylum and class level, the changes in bacterial taxa are neither disease-specific nor consistent among different studies, but a more concordant trend was observed at family and genus level.^39^

Additionally, α-diversity was thought to be a good indicator of health and diseases, and has been frequently investigated in GM analysis.^105^ However, we found that neither AD studies nor SCRD studies showed concordant variation of GM α-diversity. And α-diversity analysis was not included in several studies. This is supported by another review which examines the association between GM and PD. They found that the confounding results of α-diversity alteration reported by different studies did not substantiate the role of α-diversity analysis as reliable methods for identifying PD and its progression, suggesting that higher α-diversity was not necessarily a predictor of better health.^6^

### Future studies need to focus more on metabolic and functional analysis

Most studies examining GM alterations in AD or SCRD only evaluated compositional changes of GM, and few conducted function-related analyses such as Kyoto Encyclopedia of Genes and Genomes (KEGG) test or metabolite screening. However, reviews have indicated that two taxonomically distinct bacterial taxa could share similar functions, while two closely related taxa may act antagonistically.^92,106^ This suggests that phylogenetic analysis which is based on the hypervariable regions of bacterial 16s RNA gene cannot alone represent GM alterations at both taxonomic and functional level. It is possible that an increase of one genus could be neutralized or even reversed by a decrease of predominant genus in the same family. Thus, it would be confusing and misleading to simply conduct compositional analysis in discussing GM alterations. Moreover, metabolic and functional analysis have provided some important molecular and signaling pathways including possible interaction mechanisms between SCRD and GM and how GM dysbiosis could contribute to AD development.^10,28,30,33^

### Controversial roles of specific bacterial taxa

Lachnospiraceae and *Akkermansia muciniphila*, two taxa frequently investigated by the abovementioned studies, still remain controversial in their functions. As a core component of mammalian GM, Lachnospiraceae acts as a double-edged sword in health and disease.^92^ On the one hand, several members of Lachnospiraceae like *Blautia, Coprococcus* and *Roseburia* are crucial producers of butyrate and acetate, which induce anti-inflammatory responses, modulate insulin and lipid metabolism, and serve as the main nutrition source for colonic epithelial cells.^107–109^ But on the other hand, other members, especially those capable of both producing propionate and degrading mucin, such as *Dorea* spp, *Ruminococcus gnavus* and *Ruminococcus torques*, have been associated with series of inflammation-related disorders and increased gut barrier permeability.^93,94^ Unfortunately, the phylogenetic analyses in most studies were limited to the family level, possibly leading to the inconsistent data regarding the role of Lachnospiraceae in health and disease.

*Akkermansia muciniphila* (*A. muciniphila*) is another important SCFA-producer that utilizes mucin as carbon source.^110^ However, reduced abundance of *A. muciniphila* has been associated with inflammatory bowel diseases and elevated inflammation.^85^ Several reviews have also suggested *A. muciniphila* as a promising probiotic in treating metabolic disorders and modulating immune responses.^111,112^ Different from other mucin-degrading taxa, *A. muciniphila* was also found to promote mucin production, despite its ability to breakdown mucus layer.^113^ Nevertheless, increased level of *A. muciniphila* was found in PD patients and some opposite effects have been reported.^6,85^

### Controlling variables in human studies

At compositional level, a weak connection of GM changes between human and animal studies can be established since human and murine harbor similar yet distinct microorganisms, although a shared trend of GM alterations was observed at functional level. However, compared to human, animal models exhibited more consistent GM alterations in both AD and SCRD studies. This discrepancy is mainly due to the limited studies available, heterogeneous samples and different methodologies applied in human studies.

In animal studies, mice and rats were born with identical genetic background, housed in constant environment and fed with unified food, and variables that could compromise the study have been carefully controlled as possible. Whereas in human studies, multiple factors including race, nationality, culture background and education may have substantial impacts on the lifestyle, daily diet and eating habit of participants, which directly affect GM composition.^114^ For example, participants of the five AD patients studies we have discussed above were from three continents with diverse culture background. It has been reported that diet plays a fundamental role in health and is a key determinant of GM.^115,116^ Western-style diet, high in animal protein, sugar and fat and low in vegetables, favors the growth of Bacteroidetes, especially *Prevotella*, which has been associated with colon cancer and several bowel diseases.^117^ Mediterranean diet, featured by fruit, plant fiber and unsaturated fat, shifts GM toward more abundant *Akkermansia, Bifidobacterium* and *Lactobacillus*.^117^ Also, food rich in dietary fiber and carbohydrates promotes the growth of highly fermentative bacteria such as Lachnospiraceae, Lactobacillaceae and Ruminococcaceae in the phylum Firmicutes.^92^ Thus, the diverse dietary could contribute to the discrepant GM alterations in AD patients from different countries. Moreover, the varied experimental designs and heterogeneous methods, including fecal sample acquirement, DNA extraction and sequencing, as well as the criteria in determining cognitive function and sleep quality, make it difficult to conclude a consistent trend of GM alterations from different studies.

Therefore, it seems improper to compare GM alterations in human studies solely based on low-level phylogenetic analysis, which can be easily affected by the abovementioned factors. However, we observed a coherent trend by taking the perspective of metabolism and functions (Table 5, Figure 4).

## Conclusion

Based on the evaluations from different studies on GM at both compositional and functional levels, this review suggests a possible link between SCRD and AD by GM. We propose that long-term SCRD may indirectly lead to chronic GM dysbiosis by altering eating habit, lifestyle, metabolism, etc. SCRD and GM dysbiosis could work synergistically to contribute to the onset and progression of AD (Figure 5). However, the contribution of this alternative pathway in the development of AD remains unclear and requires further elucidation, since the etiology of sporadic AD varies from person to person.^118^ Also, more studies are needed to further demonstrate the specific mechanisms of how SCRD leads to GM dysbiosis and how probiotic and antibiotic treatment ameliorate AD pathology, as well as the potential implications of pathobionts such as Erysiopelotrichaceae and Coriobacteriaceae in health and disease.
